# The Relationship Between Zebrin Expression and Cerebellar Functions: Insights From Neuroimaging Studies

**DOI:** 10.3389/fneur.2020.00315

**Published:** 2020-04-22

**Authors:** Yi-Cheng Lin, Chih-Chin Heather Hsu, Pei-Ning Wang, Ching-Po Lin, Li-Hung Chang

**Affiliations:** ^1^Taipei Municipal Gan-Dau Hospital, Taipei, Taiwan; ^2^Department of Neurology, Taipei Veterans General Hospital, Taipei, Taiwan; ^3^Institute of Neuroscience, School of Life Sciences, National Yang-Ming University, Taipei, Taiwan; ^4^Department of Biomedical Imaging and Radiological Sciences, National Yang-Ming University, Taipei, Taiwan; ^5^Brain Research Center, National Yang-Ming University, Taipei, Taiwan; ^6^Education Center for Humanities and Social Sciences, School of Humanities and Social Sciences, National Yang-Ming University, Taipei, Taiwan

**Keywords:** zebrin, cerebrocerebellar circuits, neuroimaging, cerebellar disorders, functional topography

## Abstract

The cerebellum has long been known to play an important role in motor and balance control, and accumulating evidence has revealed that it is also involved in multiple cognitive functions. However, the evidence from neuroimaging studies and clinical observations is not well-integrated at the anatomical or molecular level. The goal of this review is to summarize and link different aspects of the cerebellum, including molecular patterning, functional topography images, and clinical cerebellar disorders. More specifically, we explored the potential relationships between the cerebrocerebellar connections and the expression of particular molecules and, in particular, zebrin stripe (a Purkinje cell-specific antibody molecular marker, which is a glycolytic enzyme expressed in cerebellar Purkinje cells). We hypothesized that the zebrin patterns contribute to cerebellar functional maps—especially when cerebrocerebellar circuit changes exist in cerebellar-related diseases. The zebrin stripe receives input from climbing fibers and project to different parts of the cerebral cortex through its cerebrocerebellar connection. Since zebrin-positive cerebellar Purkinje cells are resistant to excitotoxicity and cell injury while zebrin-negative zones are more prone to damage, we suggest that motor control dysfunction symptoms such as ataxia and dysmetria present earlier and are easier to observe than non-ataxia symptoms due to zebrin-negative cell damage by cerebrocerebellar connections. In summary, we emphasize that the molecular zebrin patterns provide the basis for a new viewpoint from which to investigate cerebellar functions and clinico-neuroanatomic correlations.

## Introduction

The cerebellum was historically regarded as a pure motor control system. However, in the past several decades studies from functional imaging (1–3) and clinical (4) studies have revealed that it is also involved in cognitive function. It remains unclear how the cerebellum relates to cognitive functions. Our interest is to integrate different cerebellar studies based on molecular strip patterns, especially the zebrin patterns (5), and we explored what was known about its connection to neuroanatomy, functional neuroimaging studies, and clinical cerebellar disorders. We would like to raise awareness of recent cerebellar studies and attempts to establish clinico-neuroanatomical correlations. Here, we reconsider the importance of molecular characteristics in cerebellar functions.

### Gross Anatomy of Cerebellum

**Figure 1:**
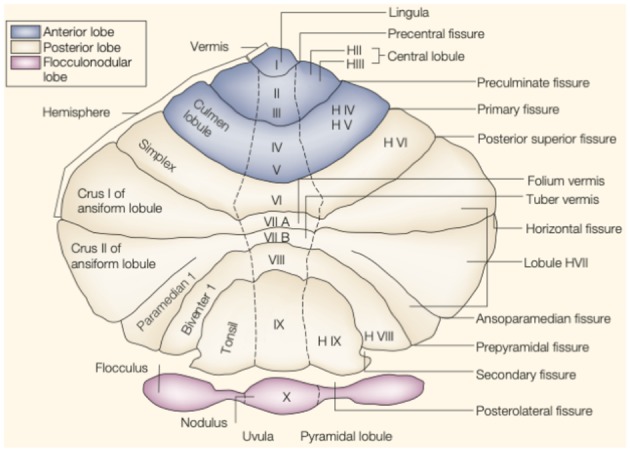
The anatomy of the cerebellum. This is an unfolded view of the cerebellar cortex showing fissures and lobules from I to X. Adapted from Manni and Petrosini (7).

The cerebellum is located in the small posterior cranial fossa, a small space in which pons and medulla oblongata are also located. The cerebellum accounts for 10% of the total mass of the brain. However, this tiny structure contains a much greater number of neurons than the much larger cerebrum (6). The gross anatomy of the cerebellum is divided into two hemispheres and one vermis. Based on surface anatomic fissures, the cerebellum consists of the anterior lobe, the posterior lobe, and the flocculonodular lobe. The anterior lobe and posterior lobe are separated by the primary fissure. Several anatomical fissures divide the cerebellar lobes into 10 smaller cerebellar lobules, from lobule I to X [Figure 1; (7)].

### Functional Subdivisions

The cerebellum can also be divided into 3 different parts based on its inputs from other brain regions: the vestibulocerebellum, the spinocerebellum, and the cerebrocerebellum. The vestibulocerebellum is phylogenetically the oldest part in the evolution of primates. The vestibulocerebellum (or flocculonodular lobe anatomically) mainly receives input from the vestibular nuclei, which are located in the medulla and pons, and projects back to the vestibular nuclei, controlling balance and ocular movements. The spinocerebellum, including the vermis, and paravermis of the hemispheres, receives sensory input mainly from the spinal cord (the spinocerebellar tract) and visual and auditory systems and projects back to deep cerebellar nuclei; based on interactions with the cerebral cortex (via the midbrain and thalamus) and the brain stem, it controls trunk and body movements. The cerebrocerebellum is the phylogenetically youngest part of the cerebellum and includes most parts of the cerebellar hemispheres. It receives input exclusively from the cerebral cortex based on cortico-ponto-cerebellar circuits and the olivocerebellar tract (via the climbing fibers). These climbing fibers project to the deep dentate nucleus and then the cerebral cortex (via the red nucleus and thalamus). The cerebrocerebellum modulates smooth and precise limb movements (7, 8).

### Cellular Anatomy

At the microscopic level, the cytoarchitecture of the cerebellum is mainly divided into three layers: the molecular, Purkinje, and granular layers. The molecular layer contains GABAergic inhibitory interneurons that form synapses onto Purkinje cell dendrites. The Purkinje layer consists of Purkinje cells, the primary integrative neurons (inhibitory GABAergic neurons) and the only source of efferent fibers from the cerebellar cortex to the deep cerebellar nuclei (9). The granule cell layer, which includes granule cells, brush cells, and Golgi cells, contains most of the neurons in the cerebellum. The granule cells are the only excitatory (glutamatergic) cells in the cerebellar cortex and send parallel fibers into the superficial molecular layer.

The afferent inputs to the cerebellar cortex consist of mossy and climbing fibers. Mossy fibers carry sensorimotor information from the ipsilateral vestibular nuclei, ipsilateral spinal cord, and contralateral pontine nuclei from cortico-pontocerebellar tracts and enter the granular layers. The climbing fibers carry sensorimotor inputs from the contralateral inferior olivary nucleus and project to and modulate the cell-firing activities of Purkinje cells. The Purkinje cell fiber projects to the deep nuclei, which is the only output from the cerebellum and controls the ultimate effect of cerebellar function. Each Purkinje cell receives input from one to seven climbing fibers, in contrast to the multiple inputs from parallel fibers and mossy fibers. The climbing fiber is a specific projection to a few Purkinje cells compared to mossy fiber, which has non-specific connections with multiple Purkinje cells (10, 11).

### Zebrin Patterns

**Figure 2:**
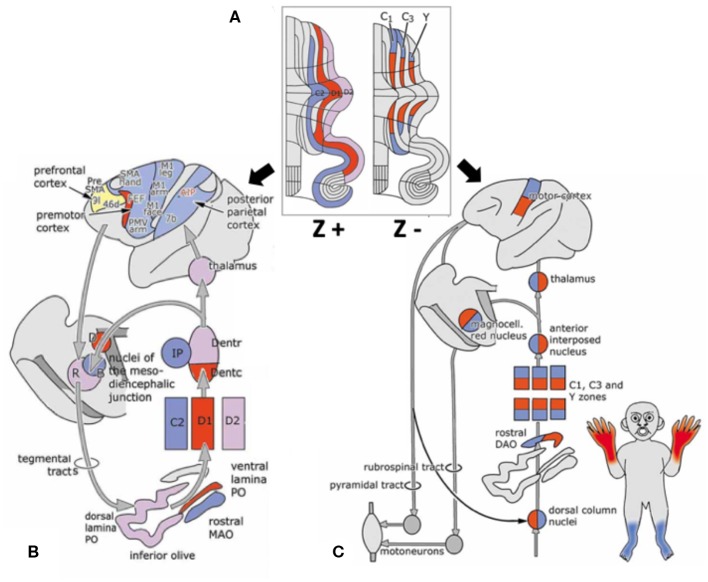
Longitudinal zebrin stripes and its cerebrocerebellar pathway. **(A)** This is a flattened cerebellar cortex showing zebrin stripes. Zebrin is a glycolytic enzyme expressed in cerebellar Purkinje cells. This figure shows the parasagittal stripes, a stereotyped array of transverse zones with an immunostaining reactive and non-reactive pattern. **(B)** The zebrin-positive zones (C2, D1, and D2 zones) are non-motor regions of the cerebellum. **(C)** The zebrin-negative zones (C1, C3, and Y zones) are motor regions of the cerebellum that receive somatosensory input. Each of the zones innervate different and specific olivo–cortico–nuclear pathways. Adapted from Voogd (17). A, A zone; B, B zone;C1–3, zones C1–3; D, dorsomedial cell column; D1,2, zones D1,2; IP, posterior interposed nucleus; MAO, medial accessory olive; PO, principal olive; VII–X, lobules VII–X of Larsell; X, X zone.

Interestingly, a striped pattern is formed by the Purkinje cell-specific antibody molecular marker zebrin (also called aldolase C) (12), which is a glycolytic enzyme expressed in cerebellar Purkinje cells (13, 14). This aldolase C enzyme expresses specifically in the hippocampus and Purkinje cells and plays an important role in ATP biosynthesis (15). Hawkes et al. demonstrated parasagittal stripe, a stereotyped array of transverse zones with zebrin immunostaining reactive and non-reactive patterns. The zebrin stripe constitute seven longitudinal bands identified by Professor Voogd, who used an acetylcholinesterase stain in the cerebellar cortex (16, 17). The zebrin-negative zones (C1, C3, and Y zones) are motor regions of the cerebellum that receive somatosensory input, and the zebrin-negative Purkinje cells fire at higher frequencies. On the other hand, the zebrin-positive zones (C2, D1, and D2 zones) are non-motor regions of the cerebellum, and the zebrin-positive Purkinje cells fire at lower frequencies (18). Each zone innervates different and specific olivo-cortico-nuclear pathways [Figure 2; (19)]. Larson et al. showed that electrical stimulation of nerves from different limbs have distinct climbing fiber responses in different longitudinal zones (20). In other words, the climbing fibers and those corticonuclear projections define these zebrin patterns, which may be the basic functional units of the cerebellum (21, 22). In addition, Richard et al.'s “one-map” hypothesis describes that climbing fibers have a closed-loop projection to specific zebrin patterns and unify the cerebellar map from the perspective of anatomy, embryology, and physiology (23). In sum, the zebrin-positive/-negative longitudinal patterns have different and specific connections and are related to distinct functions.

The functions of these zebrin patterns are potentially related to long-term potentiation (LTP) and long-term depression (LTD) (24). LTP produces a long-lasting increase in neuron activity based on the recent activation history of the cell, which is a well-characterized form of synaptic plasticity, especially in memory. In contrast, LTD produces a long-lasting decrease in synaptic strength, which is important for motor learning (25). LTD in zebrin-negative Purkinje cells is easier to induce than in zebrin-positive Purkinje cells (5, 25). This may represent the predominant form of plasticity in zebrin-positive zones (26), which is correlated with the non-motor function of the cerebellum. In summary, not only zebrin-positive/-negative cells and their specific connections, but also the physiological capacities of the zebrin cells may be a fundamental contributor to the cerebellar system. We consider that the zebrin patterns may be the cornerstone of the cerebellar functions and related to the clinical presentation of cerebellar disorders.

## Cerebellar Functional Mapping from Neuroimaging Studies

It is difficult to trace the projection from the cerebral cortex to the cerebellum in the cortico–ponto–cerebellar pathway because the cerebrocerebellar connections are indirect and polysynaptic. Currently, the sole *in vivo* method to delineate neuronal pathways is tractography, based on diffusion-weighted imaging (27). However, the polysynaptic cerebrocerebellar pathways, which have contralateral connections, pass through the cerebellar deep nucleus and penetrate into the heavily folded cerebellar cortex; these factors make cerebrocerebellar circuits difficult to trace *in vivo* (28–31). Even so, *ex vivo* studies using retrograde transsynaptic tracers (rabies virus or herpes simplex virus) have identified a few cerebrocerebellar connections, including cerebello–thalamo–cortical and cortico–ponto–cerebellar pathways, and have shown that the primary motor cortex (M1) is linked with cerebellar lobules III–VI and VIII whereas dorsolateral prefrontal cortex area 46 is linked to crus II and lobule X (32, 33). This evidence suggests that cerebrocerebellar circuits are involved in sensorimotor control and higher cognitive functions such as attention, executive control, language, working memory, learning, pain, emotion, and addiction (34, 35).

Because the cerebellum was initially considered to be responsible for only motor control and its complicated polysynaptic nature, only a few studies made connections between cognition and the cerebellum. For example, a notable exception was Petersen et al., who used positron emission tomography (PET) in 1988 to demonstrate that crus I and crus II in the right cerebellum are involved in the linguistic single-word processing of verbs when hearing some objects, such as “drink” water (36). More imaging studies about the latest neuroimaging techniques and findings related to the cerebellum are discussed next.

### Resting-State fMRI Studies of Cerebellum

**Figure 3:**
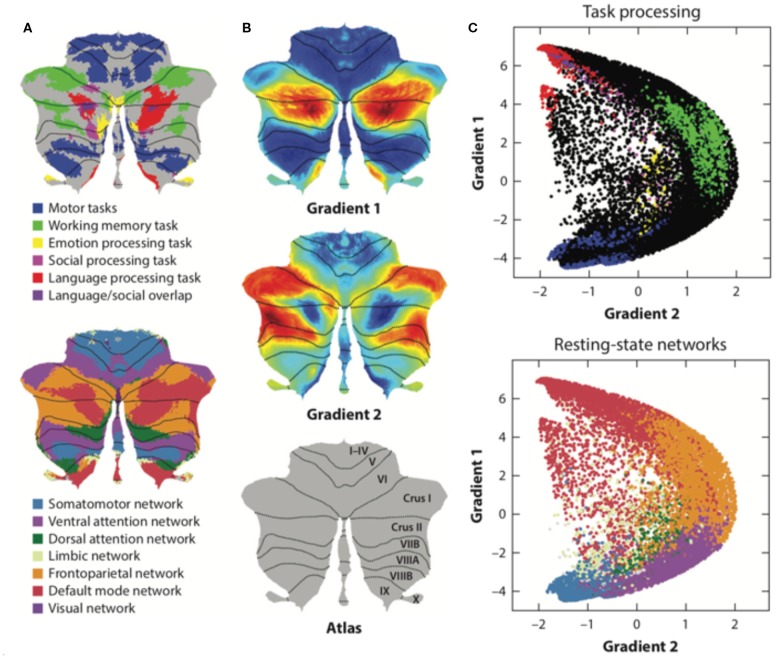
The functional maps and functional gradient of the cerebellum. **(A)** Resting-state functional fMRI shows cerebellar functional topography and is correlated with different non-motor networks, such as somatomotor, fronto-parietal, dorsal attention, ventral attention, limbic networks, salience network, executive control circuitry, and the default-mode network. Task-evoked fMRI research has shown that lobule V is activated for sensorimotor tasks; VIIIA/B for motor tasks; VIIIB for somatosensory activation; lobule VI and crus I for language and verbal working memory; lobule VI for spatial tasks; lobules VI, crus I, and VIIB for executive functions; and lobules VI, crus I, and medial VII for emotional processing. **(B,C)** Axis I extends from the primary motor to transmodal regions with the primary–unimodal–transmodal hierarchical principle. Axis II isolates the working memory/frontoparietal network areas and extends from task-unfocused to task-focused processing. The results follow a gradual organization of the well-established cerebellar distributions by using the functional gradient method. Adapted from Buckner et al. (2), Guell et al. (37), and Schmahmann et al. (38).

Resting-state functional magnetic resonance imaging (fMRI) is commonly used to study functional topography. In particular, resting-state functional connectivity (FC) fMRI has revealed a relationship between the cerebellum and several non-motor brain networks, including the somatomotor, frontoparietal, dorsal attention, ventral attention, limbic, salience, executive control, and default-mode networks (2). A unique cerebellar functional topography was demonstrated, with different regions being correlated with different non-motor networks [Figure 3A; (2, 39)]. The sensorimotor cerebellum involves in the anterior lobe, in lobule VIII and part of lobule VI, whereas the cognitive cerebellum involves in the posterior lobe (especially crus I and crus II) and vermis (40). In connectivity studies, the intrinsic connectivity networks (ICNs), the functional coupling between the distant brain cortex and the cerebellum, showed network mapping, including motor networks in the anterior lobe and lobule VIII and cognitive networks (dorsal attention, ventral attention, frontoparietal, default-mode, and salience networks) in the posterior lobe (2, 3, 39).

However, both cognitive and sensorimotor clusters are present within lobule VI. The sensorimotor network clusters are located more centrally and closer to the paramedian part of the cerebellum. The cognitive network clusters are located more laterally and closer to the post-erosuperior fissure (39, 40). More recent studies have demonstrated that lobule VI is a hub controlling sensorimotor and motivations. Lobule VI is an integrative interface between motor and cognitive/emotional circuits during a motor task with verbal encouragement. This hub controlling function may explain the overlap of both cognitive and sensorimotor clusters in lobule VI in fMRI studies.

### Task-Evoked fMRI Studies of Cerebellum

Task-evoked fMRI detected blood-oxygen-level dependent (BOLD) signals changes in cerebellum when different tasks performed, such as sensorimotor tasks, language tasks, verbal working memory tasks, spatial tasks, executive function tasks, and emotional processing tasks. Task-evoked fMRI studies have shown that lobule V is activated during sensorimotor tasks; VIIIA/B during motor tasks; VIIIB during somatosensory tasks; lobule VI and crus I during language and verbal working memory tasks; lobule VI during spatial tasks; lobule VI, crus I, and VIIB during executive function tasks; and lobule VI, crus I, and medial VII during emotional processing (1). The functional lateralization of the cerebellum was noted in task-evoked fMRI studies (for example, the language task was linked to the right side of cerebellar lobule VI and crus I, and the spatial task was linked to the left side of cerebellar lobule VI), indicating that the information processing pathways crossed hemispheres between the cerebral cortex and cerebellum (1). However, the motor control system of the cerebellum is “double-crossed” (which is different from the single-crossed non-motor system in the cerebellum): The first crossing occurs in the decussation of the superior cerebellar peduncle while the second crossing occurs in the corticospinal or rubrospinal tracts descending into the spinal cord. Therefore, the cerebellar hemisphere modulates ipsilateral limb movements.

Moreover, social cognition tasks, including theory of mind (mirroring, event mentalizing, person mentalizing, abstraction) (41) and emotional affective processing (a painful experience of our own or from others), have also been examined in fMRI studies (40, 42). In these studies, a theory of mind task activated crus I in the bilateral cerebellum; an emotional affective processing task showed that the posterior cerebellar vermis was related to painful first-person experiences whereas the posterior cerebellar hemisphere (lobule VI) was related to empathetic pain on behalf of others (Figure 3A). In summary, cerebellar functional topography was revealed by multiple task-evoked MRIs with both cerebrum and cerebellum activation. This result emphasizes the role of the cerebellum in both motor and non-motor functions as well as its cerebrocerebellar connections. All these cerebrocerebellar connections project to different Purkinje cells. Further studies should focus on these cerebrocerebellar pathways and their relationships to zebrin patterns.

### Functional Gradient Neuroimaging Studies of Cerebellum

Functional and anatomical structure matches are crucial for the definition of cerebellar functional neuroanatomy, which means that anatomy reflects a functional hierarchy from primary to transmodal processing (43). Recently, Guell et al. demonstrated a novel functional imaging technique. This new method applies diffusion maps embedded into the brain's resting-state imaging results followed by two principal gradients of resting-state FC in the cerebral cortex. Axis I extends from the primary motor to transmodal regions with the primary–unimodal–transmodal hierarchical principle (43). Axis II isolates working memory/frontoparietal network areas and extends from task-unfocused to task-focused processing. The results follow a gradual organization of the well-established cerebellar distributions by using the functional gradient method [Figures 3B,C; (37)]. The gradients were interpreted by analyzing their relationship to resting-state and task-evoked fMRI cerebellar maps (37). When each cerebellar anatomical voxel was arranged along the two functional axes, a gradual pattern of organization emerged.

Even so, the functional maps (including resting fMRI and task-evoked fMRI) do not necessarily align with the anatomical lobules. Recently, King et al. compared the similarity of paired voxels within a region across a anatomical boundary using a multidomain task battery in fMRI and revealed that, although functional boundaries existed, they were not aligned with either anatomic lobules or zebrin stripe (44). Bernard et al. also demonstrated that the lobular boundaries were not necessarily indicative of functional boundaries by comparing the anatomical and self-organizing map approaches in the resting-state cortico-cerebellar FC networks (3). These neuroimaging studies also support the “one-map” hypothesis by unifying the cerebellar map not only anatomically but also functionally (23).

## The Role of Cerebellum in Neurological Diseases

Next, we review studies of diseases related to cerebellar dysfunction, with a particular focus on clinical ataxiology and its contributing clinical expression of cerebellar pathology: cerebellar motor syndrome, vestibular cerebellar syndrome, and cerebellar cognitive affective syndrome (CCAS) (45). CCAS, also called Schmahmann's syndrome, was first described in 1998; it presents with neuropsychiatric symptoms, such as the blunting of affect, lack of initiation, apathy, depression, and loss of empathy or disinhibited, irritable, and inappropriate behavior (4, 46). These studies provide evidence that underlies the complexity of cerebellar symptoms despite the undergoing disease and our understanding of cerebellar function, through the various forms of pathophysiology they encompass.

### Cerebellar Lesions

Cerebellar stroke patients typically present with dizziness, ataxia, dysmetria, and postural imbalance. However, atypical cerebellar stroke patients may present with only dizziness or CCAS and without obvious cerebellar symptoms or signs by neurological examinations, which are difficult to diagnose. Moreover, these cerebellar strokes lead to notably poor outcomes (47). Following cerebellar stroke, cerebellar motor syndrome was shown to be associated with the anterior lobe, while CCAS was associated with the posterior lobe in a voxel-based lesion symptom mapping study analyzing relationships between behavioral deficits in neurological populations and lesion sites associated with those deficits (48). Cerebellar mutism syndrome (CMS) was also noted in children after surgery for tumors in the posterior fossa, which presented as mutism, emotional lability, hypotonia, and ataxia (49, 50). Patients with tumor compression to the cerebellum, such as posterior fossa arachnoid cyst or Chiari malformation, also presented with neurodevelopmental and psychiatric symptoms (developmental delay, intellectual disability, autistic, and obsessive-compulsive symptoms) (51).

In summary, cerebellar lesions located in the anterior lobe and parts of lobule VI interrupted cerebellar communication with cerebral and spinal motor systems and caused cerebellar motor syndrome. Cerebellar lesions located in the posterior lobe (lobules VI and VII) interrupted the cerebellar modulation of cerebrocerebellar cognitive loops and caused cognitive impairments. Finally, cerebellar lesions located in the vermis interrupted cerebrocerebellar limbic loops and caused neuropsychiatric symptoms (40).

### Spinocerebellar Ataxias

Spinocerebellar ataxias (SCAs) are rare inherited neurodegenerative cerebellar disorders with clinical and genetic heterogeneities. The most clinically significant symptoms of SCAs are ataxia, dysmetria, dysarthria, and oculomotor signs (52, 53). The prevalence of SCA subtypes varies across populations (54–56). SCA1, SCA2, and SCA3 are the most common subtypes in Caucasians, while SCA2, SCA3, and SCA6 are more frequently encountered in Japanese and Chinese people (57, 58). Nonataxia symptoms, including cognitive impairment, neuropsychiatric symptoms, and social cognition deficits, have been found in SCA patients (59–62). Cerebellar volume decreases have been noted in SCA patients. SCA1, SCA2, and SCA3 patients presented with atrophy in the cerebellar hemispheres and vermis as well as the brainstem. Atrophy in the cerebellar hemispheres was less severe in SCA3 patients than in SCA1 and SCA6 patients. However, atrophy in those with SCA6 was restricted to the pure cerebellar cortex without vermis and brainstem (63, 64). Functional MRI connectivity studies in SCA2 and SCA3 patients showed decreased connectivity between the sensorimotor area in the cerebral cortex and the cerebellum (65, 66). In SCA6 patients, cerebellar lobules V and VI and the dentate nuclei were more active compared to controls when performing a hand-movement task (67). DTI studies in SCA6 patients also showed increased connectivity between the cerebral cortex (especially the occipital cortex) and cerebellum in moderate cases and decreased connectivity in severe cases (68). This result may indicate a compensatory phenomenon in the cerebrocerebellum circuit in response to spinocerebellum dysfunction in SCA6 patients (69). Indeed, patients with SCA6 progress slower than those with SCA1, SCA2, SCA3, and SCA17 and have longer disease durations (70). The chronic nature of SCA6 may be related to compensatory phenomena involving increased cerebrocerebellar connections during cerebellar degeneration.

Recently, Hashimoto and Honda et al. demonstrated different internal model disruptions in aging people and spinocerebellar ataxia patients (SCA6 and SCA31) by using prism adaptation tasks (71, 72). These studies also established links between the internal models and the clinical presentation of cerebellar disorders. More studies are needed to investigate the relationship between clinical presentation and internal models.

### Neurodegenerative Disease and the Cerebellum

Alzheimer's disease (AD) is the most common form of dementia. The deposits of amyloid plaques and ubiquitin-immunoreactive dystrophic neurites are found in not only the cerebral cortex, but also the cerebellum (73, 74). Decreased volumes of cerebellar gray matter have been found in patients with early-onset AD (75). However, whether there are changes in the FC of the cerebrocerebellar connections in patients with AD and mild cognitive impairment (MCI) remains a controversial question. Some resting-state fMRI studies have shown decreased FC of the cerebrocerebellar connections in MCI and AD patients (76, 77). However, in another study, decreased FC of the cerebrocerebellar connections in resting-state networks was found in the AD group whereas a significant increase in FC was noted in the MCI group (78). This connectivity difference indicated a compensatory phenomenon in the cerebrocerebellum connection in the early stages of AD; in other words, these controversial results may have been due to connectivity changes during the clinical progression of AD. Future studies could focus on subgroup analyses based on the clinical cognitive impairment severity to investigate the cerebrocerebellum connectivity differences at each of the different stages of disease progression (69, 78). In addition, a syndrome with cognitive impairment and dynapenia, called physio-cognitive decline syndrome (PCDS), is considered to be correlated with the cerebellum (79, 80). Chen et al. demonstrated reduced gray matter volume (GMV) in the cerebellum, hippocampi, middle frontal gyri, and several other cerebral regions in the prefrail and frail groups compared to the robust group (81).

Parkinson's disease (PD) is also a common neurodegenerative disorder associated with motor and cognitive impairments. The main pathophysiology of PD is the degeneration of dopaminergic neurons in the striato–thalamo–cortical pathways. The anatomical connections between the basal ganglia and cerebellum have been elucidated (82). Cerebellar atrophy has been noted in PD and Parkinson plus syndromes [including multiple system atrophy (MSA) and progressive supranuclear palsy (PSP)] (83–86). Connectivity DTI studies have shown decreased FA in the cerebellar hemispheres in PD patients (87). However, increased connectivity in the cerebellum, primary sensorimotor cortex, and premotor area was found when using a regional homogeneity (ReHo) method in fMRI (88). In addition, enhanced connectivity between the dentate nucleus and cerebellum in PD patients was noted in resting-state fMRI (89). The reasons for the increased connectivity observed in these studies are still unknown, but the findings suggest that cerebellar involvement in PD is compensatory (90).

### Multiple Sclerosis and the Cerebellum

Multiple sclerosis (MS) is an inflammatory disease with clinical mono- or polysymptomatic presentations depending on the location of the demyelinating lesion. In some cases, there are asymptomatic lesions as well-symptoms without corresponding lesions. The most common presentations are optic neuritis, cerebellum, brainstem, and spinal cord syndromes (91). The cerebellum is one of the most commonly involved brain regions in MS patients, with cerebellar lesions being described in approximately half of all cases in clinically defined MS (92). Cerebellar structural atrophy in MRI studies has been noted in MS patients (93). D'Ambrosio et al. demonstrated that cerebellar volumetric abnormalities were correlated with the clinical symptoms and motor and cognitive performance impairments in MS patients. Lower anterior cerebellar volume and brain T2 lesion volume predicted worse motor performance, whereas lower posterior cerebellar volume and brain T2 lesion volume predicted poor cognitive performance (94), which maps into the previously described functional cerebellar topography (40). In addition, resting-state fMRI in progressive MS showed reduced FC between crus II and the right frontal pole and increased FC between lobule VIIb and the right precentral gyrus after controlling for structural damage (95). Sbardella et al. also showed enhanced dentate FC to frontal and parietal cortical areas in MS patients compared to healthy controls, and the increased connectivity was related to better cognitive performance (96). These studies indicate the plasticity of cerebrocerebellar circuits and functional compensation when damage occurs (69, 97).

In summary, the clinical presentation of ataxia is typically taken as the first sign of cerebellar disease, but neuropsychiatric impairments are often not diagnosed at that stage—perhaps in part because the cerebellum is still perceived by some physicians as being responsible purely for motor control, meaning the relevance of non-motor symptoms to the cerebellum might be overlooked. A second reason for the lack of early diagnosis may be the lack of neuropsychiatric assessment tools for CCAS. Recently, Schmahmann developed a scale for evaluating the clinical neuropsychiatric symptoms in patients with cerebellar disorders, including patients with Mini-Mental State Examination (MMSE) scores >28 (i.e., normal cognitive function). The Schmahmann's syndrome scale includes tests of executive function, language, visual spatial function, and affect regulation with sensitivity and selectivity for detecting patients with CCAS of 85%/74% in exploratory cohorts and 95%/78% in validation cohort studies (98). By using this CCAS scale, we can clinically identify patients with cerebellar non-motor symptoms. The third reason could be that ataxia and dysmetria symptoms present earlier than non-ataxia symptoms. Interestingly, neuroimaging studies in AD, PD, MS, and SCA patients showed compensatory phenomena (69, 88, 89, 97). We hypothesized that these compensatory phenomena are related to zebrin patterning based on the evidence from molecular, functional imaging, and clinical studies (see next).

### Combination of Multiple Neuroimaging Studies of the Cerebellum and Zebrin

#### Compensatory Phenomena and Zebrin

We hypothesized that zebrin-positive cells contribute to the compensatory phenomena observed in cerebellar-related chronic neurodegenerative diseases. In particular, zebrin-positive cerebellar Purkinje cells are resistant to excitotoxicity, cell injury, and degenerate slowly (99), the brain has time to compensate for disrupted cerebral function by increasing the cerebrocerebellar connectivity. Indeed, several neuroimaging studies have shown enhanced cerebrocerebellar connectivity in chronic neurodegenerative diseases (such as SCAs, AD, PD, and MS) (69, 88, 89, 97). The compensatory phenomena in the cerebrocerebellar circuit indicate the potential role of plasticity in the cerebellum.

This hypothesis can also explain why the ataxia and dysmetria symptoms present earlier than non-ataxia symptoms and the neuropsychiatric impairment symptoms of CCAS are less severe than the ataxic symptoms. Because zebrin-positive cells have been associated with the non-motor cortical regions and zebrin-negative cells have been associated with motor cortical regions (17). We suggest that motor control dysfunction symptoms such as ataxia and dysmetria present earlier and are easier to observe than non-ataxia symptoms due to zebrin-negative cell damage. Cerebellar zebrin-positive cells are resistant to cell injury (99) while zebrin-negative zones are more prone to damage (12, 18), suggesting that zebrin-positive Purkinje cells and its cerebrocerebellar connection are preserved with normal non-motor functions when brain damage occurs. We believe that the clinical presentations of these diseases and neuroimaging studies might be connected with cerebellar anatomical zebrin patterns, suggesting a new way to investigate these cerebellar disorders.

### Triple Functional Representation of the Cerebellum

**Figure 4:**
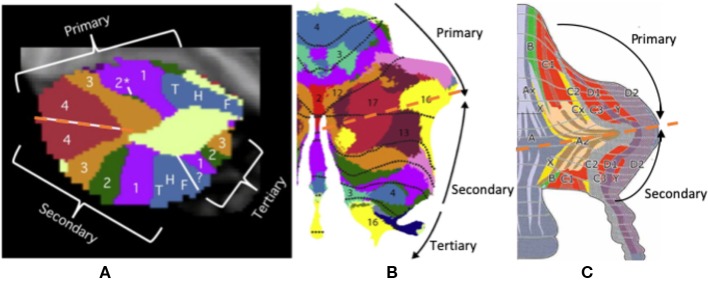
The triple presentation of cerebellar functional topography. **(A,B)** This figure shows the triple functional representation topography in the cerebellum: primary, secondary, and tertiary representation. The primary and secondary representations show a mirror-symmetry order of the functional topographic maps of language, working memory, and social and emotional processing by task-evoked and seed-based resting-state fMRI. **(C)** The symmetrical pattern of primary and secondary representation is similar to up-and-down symmetrical molecular zebrin patterns. Adapted from Voogd (17), Buckner et al. (2), and Diedrichsen et al. (100).

Triple functional representation topography of the cerebellum was demonstrated by Guell et al.—namely, primary, secondary, and tertiary representation (Figure 4). The primary and secondary representations showed a mirror-symmetry order of the functional topographic maps of language, working memory, and social and emotion processing by task-evoked and seed-based resting-state fMRI (37, 101). The symmetrical patterns of primary and secondary representation are similar to up-and-down symmetrical molecular zebrin patterns, which may indicate that those cerebellar areas with the same functions (with the same color in Figure 4) have similar zebrin cell properties. Furthermore, these symmetrical cerebellar areas carry identical inputs from cerebrocerebellar pathways with specific correspondence. For example, the symmetrical motor function representations carry inputs from the motor cortex. Interestingly, the tertiary representation was located in the vermis and para-vermis region, which are not symmetrical to primary and secondary representation. We hypothesized that three representations of the cerebellum maybe corelated to its cerebrocerebellar pathways. The functional synaptic organization of cerebrocerebellar fibers in vermis were conserved in a study with mice compared to 85% synaptic silence in the cerebellar hemisphere (102, 103). Therefore, the triple functional representation topography may be explained by an up-and-down symmetrical molecular zebrin pattern with specific cerebrocerebellar pathways as well as one vermis/paravermis region that preserves multiple functional pathways. The function of the vermis also supports its multiple pathways, which is responsible for not only body posture, locomotion, and eye movement (the so-called spinocerebellum), but also emotional behavioral changes and autonomic functions (the so-called limbic-connected cerebellum) (8, 42). This symmetrical observation implies the importance of the zebrin patterns and their correlation to functional maps. Further research might be critical for making the connection among molecular, anatomical, and functional representations.

Taking the many neuroimaging and disease studies of the cerebellum together, we noticed that the traditional human cerebellar anatomical lobules are very different from the molecular zebrin patterns and cerebellar functional topographic maps. By unifying the anatomical, molecular, and functional topography, we can explain how the cerebellum works through cerebrocerebellum circuits, affecting the various networks. Many neuroimaging studies have revealed cerebellar functional topographical features that are correlated with different brain networks related to motor and non-motor functions. These functional areas cross anatomical lobule borders (3, 44), indicating that functional maps are different from anatomical topography. Considering the similarity of three functional representations and zebrin patterns of cerebellum, the zebrin-positive/-negative cells' properties may be more crucial to cerebellar functions. Due to the current limitations of neuroimaging techniques, we cannot demonstrate zebrin stripe in conventional MRI studies. However, Boillat et al. showed similar longitudinal stripe-like patterns by using quantitative T1 and T2^*^ mapping at ultrahigh field (7T) MRI (104). Solodkin et al. also demonstrated the visualization of zebrin-like patterns in SCA1patients by fMRI connectivity and this zebrin patterns diminished during the disease progression of SCA1 (105). Therefore, by separating the zebrin-like signals in neuroimaging studies, we might be able to study the alteration and function of zebrin stripe and its relationship to clinical disease.

## Conclusions

In this article, we reviewed cerebellum-related studies from multiple viewpoints, including aspects of molecular function, structural features, functional imaging, and clinical diseases. Due to the complexity and incomplete nature of the field being reviewed, the overall picture of the mechanisms underlying cerebellar functions remains confusing and challenging. We emphasized in particular the roles of zebrin stripe in the cerebellum, which have been found to be related to specific climbing fibers and their olivo–cortico–nuclear pathways. Evidence from several neuroimaging studies has demonstrated the presentations of cerebellar functional topography, which is similar to the zebrin pattern. The cerebellar functional maps cross the borders of the conventional anatomical cerebellar lobules. Functional imaging studies have also shown compensatory phenomena in cerebellum-related diseases, such as AD, PD, MS, and SCA. We hypothesized that these compensatory phenomena are related to zebrin patterning and offered a new perspective on cerebellar functions based on the evidence from molecular, functional imaging, and clinical studies.

This review has some limitations. The precise relationships among the anatomical cerebellum, molecular stripe patterns, and functional topography are unknown. The neuroimaging studies showed zebrin-like patterns, but we need more studies to confirm the zebrin-like patterns in imaging studies are identical to the molecular zebrin patterns. The functional imaging of molecular zebrin pattern is still being developed. Furthermore, a computational model of cerebrocerebellar connections that addresses how the cerebellum processes information and regulates cerebrocerebellar network circuits is still unavailable.

Several fundamental theories of cerebellar function exist. One of the most famous theories regarding cerebellum functionality is the dysmetria of thought theory based on the universal cerebellar transform (UCT) (4, 38, 46, 106, 107). This model implies a general function for regulating multiple circuits. In this framework, impairments in UCT function cause the dysregulation of these subcircuits that leads to functional loss, which has been called “the dysmetria of thought theory” (108). However, multiple functionality theory suggests unique and diverse functions for the different circuits (100). The electrophysiology of the zebrin pattern is the key that holds the answer regarding which fundamental theory is preferable. If zebrin-positive and zebrin-negative cells have similar electrophysiological cell activity but different functions due to distinct and specific olivo–cortico–nuclear pathways, the universal cerebellar transform is supported. However, if zebrin-positive and zebrin-negative cells have different electrophysiological activities coupled with different pathways, the multiple functionality theory is more likely. We need more studies to investigate these fundamental theories.

Furthermore, the compensatory phenomena resulting in enhanced cerebrocerebellar circuits are important for plasticity and learning. Cerebellar stimulation with techniques such as rTMS or TDCS and the application of other forms of cerebrocerebellar circuit neuromodulation may be effective for the control of motor symptoms or CCAS in patients with cerebellar lesions. At present, cerebellar plasticity produced by cerebellar regulation is still a new research topic. More research is required to understand the neural mechanisms, their relationship to zebrin-positive and zebrin-negative cells (5), and their association with the neuromodulation of the cerebellum based on this knowledge with potential therapeutic applications for different cerebellar disorders (6).

## Author Contributions

Y-CL, P-NW, C-PL, and L-HC: conceptualization of the study. Y-CL, C-CH and L-HC: manuscript.

## Conflict of Interest

The authors declare that the research was conducted in the absence of any commercial or financial relationships that could be construed as a potential conflict of interest.
